# Distinct Contributions of Whisker Sensory Cortex and Tongue-Jaw Motor Cortex in a Goal-Directed Sensorimotor Transformation

**DOI:** 10.1016/j.neuron.2019.07.008

**Published:** 2019-09-25

**Authors:** Johannes M. Mayrhofer, Sami El-Boustani, Georgios Foustoukos, Matthieu Auffret, Keita Tamura, Carl C.H. Petersen

**Affiliations:** 1 Laboratory of Sensory Processing, Brain Mind Institute, Faculty of Life Sciences, Ecole Polytechnique Fédérale de Lausanne (EPFL), Lausanne, Switzerland

**Keywords:** reward-based learning, goal-directed sensorimotor transformation, whisker sensory processing, somatosensory cortex, licking, motor cortex

## Abstract

The neural circuits underlying goal-directed sensorimotor transformations in the mammalian brain are incompletely understood. Here, we compared the role of primary tongue-jaw motor cortex (tjM1) and primary whisker sensory cortex (wS1) in head-restrained mice trained to lick a reward spout in response to whisker deflection. Two-photon microscopy combined with microprisms allowed imaging of neuronal network activity across cortical layers in transgenic mice expressing a genetically encoded calcium indicator. Early-phase activity in wS1 encoded the whisker sensory stimulus and was necessary for detection of whisker stimuli. Activity in tjM1 encoded licking direction during task execution and was necessary for contralateral licking. Pre-stimulus activity in tjM1, but not wS1, was predictive of lick direction and contributed causally to small preparatory jaw movements. Our data reveal a shift in coding scheme from wS1 to tjM1, consistent with the hypothesis that these areas represent cortical start and end points for this goal-directed sensorimotor transformation.

## Highlights

• Localization of tongue-jaw motor cortex by anatomical and functional mapping
• Inactivation of tongue-jaw motor cortex inhibits goal-directed contralateral licking
• Neurons in tongue-jaw motor cortex encode licking direction but not sensory cue
• Neurons in whisker sensory cortex encode sensory cue but not licking direction

## Teaser

Mayrhofer et al. map the location of mouse tongue-jaw motor cortex, finding that its neuronal activity encodes licking direction and is necessary for contralateral goal-directed licking but does not encode which sensory cue initiates licking, in contrast to sensory cortex.

## Introduction

Animals can learn to transform sensory input into motor output in order to obtain rewards. The neuronal circuits responsible for learning and execution of such goal-directed sensorimotor transformations remain incompletely understood, with the neocortex thought to play a key role (Carandini and Churchland, 2013, Georgopoulos, 1991, Hallett, 2007). For mice, many cortical regions appear to participate, even in simple goal-directed sensorimotor transformations (Allen et al., 2017, Goard et al., 2016, Guo et al., 2014, Le Merre et al., 2018). Different areas of neocortex are specialized for distinct functions, with some being more important for sensory processing and others being more involved in controlling movement. An essential step toward mechanistic understanding of goal-directed sensorimotor transformations is to identify where and how relevant sensory and motor information is represented within cortex during task performance.

Primary sensory areas of neocortex are innervated by specific thalamic nuclei driving the processing of incoming external information. Primary sensory areas have well-defined topographic maps, for example, retinotopy in visual cortex, tonotopy in auditory cortex, and somatotopy in somatosensory cortex. Causal contributions of neuronal activity within primary sensory cortices have been ascribed in various goal-directed sensorimotor transformations (Jaramillo and Zador, 2011, Resulaj et al., 2018). The whisker system provides important tactile information for rodents, and the primary whisker somatosensory cortex (wS1) contains an anatomical map in which each whisker is distinctively represented (Woolsey and Van der Loos, 1970). Neuronal activity in wS1 has been shown to be involved in whisker-based decision-making tasks (Guo et al., 2014, Miyashita and Feldman, 2013, Sachidhanandam et al., 2013, Yang et al., 2016), thus providing a well-defined starting point for cortical processing of whisker-related information.

Motor maps are more difficult to define (Auffret et al., 2018, Brecht et al., 2004, Ferrier, 1874, Graziano et al., 2002, Matyas et al., 2010, Penfield and Boldrey, 1937). Evoked movements of specific body parts depend upon the precise nature of the intracortical stimulation and the state of the animal. An anatomical definition of motor maps would therefore be helpful. By assuming that sensory information from a given body part is of particular relevance to the motor control of that body part, we can map frontal cortex through its direct innervation from primary sensory areas. For the mouse whisker system this definition appears to work well, with an anatomical projection from wS1 to a specific region anterior and lateral to bregma providing a precise definition for the location of primary whisker motor cortex (wM1) (Ferezou et al., 2007, Kleinfeld and Deschênes, 2011, Mao et al., 2011, Matyas et al., 2010).

Licking is the required motor output to report perceptual choice in many sensory decision-making tasks for head-restrained mice. Licking involves the opening of the jaw and the protrusion of the tongue toward a specific target (Guo et al., 2014, Mayrhofer et al., 2013). Cortical contributions to licking motor control might come from the primary tongue and jaw motor cortex (tjM1). Here, we define tjM1 as the frontal region receiving input from the tongue and jaw representations in primary sensory cortex (tjS1), and, using behavioral switch tasks, optogenetic inactivation, and two-photon imaging of neuronal network activity, we test the hypothesis that wS1 and tjM1 might represent cortical start and end points of an abstract sensory-to-motor transformation in which a brief whisker deflection is converted into goal-directed licking in order to obtain reward.

## Results

### Identification of Tongue-Jaw Primary Motor Cortex

**Figure 1:**
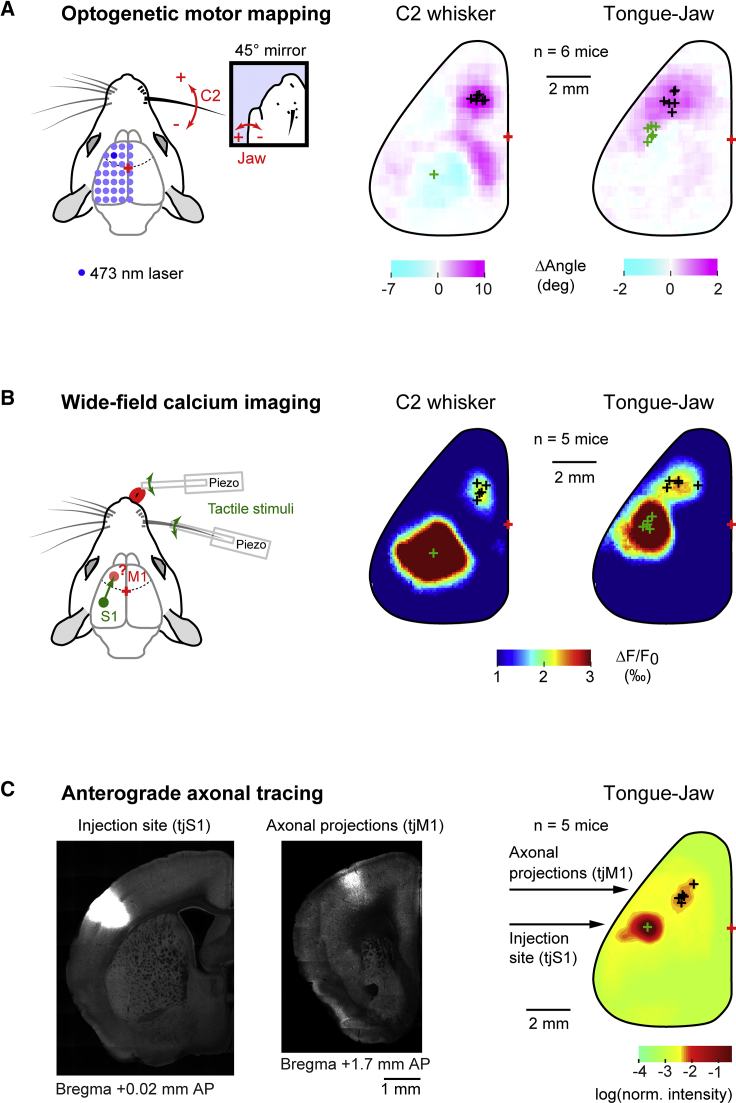
Identification of Tongue-Jaw Primary Motor Cortex (A) For optogenetic motor mapping, a blue laser beam was directed in a grid-like manner over the dorsal cortex (blue dots) of a Thy1-ChR2 mouse. Whisker and jaw movements (side view by 45° mirror) were filmed simultaneously by a high-speed camera. Grand average (n = 6 mice) motor maps for the C2 whisker and the jaw were aligned on the intrinsic optical signal for the C2 whisker. Black crosses indicate the center of the frontal cortical region that evoked movements for individual mice. Green crosses indicate centers of the intrinsic optical signal evoked by C2 whisker and tongue-jaw sensory stimulation for each mouse. Red crosses represent average bregma position. (B) For wide-field functional calcium imaging, either the C2 whisker or the tongue was stimulated with a piezoelectric actuator in Thy1-GCaMP6f mice. Grand average (n = 5 mice) sensory-evoked responses during a 100–200 ms post-stimulus time window for C2 whisker (left) and tongue-jaw (right) stimulation were aligned to the wS1 calcium signal evoked by C2 whisker stimulation. Green crosses indicate centers of the first activation spot during the early phase of the response (0–100 ms). Black crosses are the centers of the frontal secondary spot (100–200 ms) for individual mice. Red crosses represent average bregma position. (C) For anterograde axonal tracing, AAV-hSyn-turboRFP was injected in the cortical region where the first activation spot of tongue-jaw stimulation (tjS1) was detected by calcium imaging. Axonal projections were found in a localized column in motor cortex (tjM1). Grand average (n = 5 mice) of cortical fluorescence aligned to the injection site (right). Black crosses indicate centers of the anterior spot for each mouse. Green cross corresponds to the center of the injection site. Red cross represents average bregma position. See also Figure S1, Table S1, and Video S1.

Licking requires jaw and tongue muscles to be orchestrated in a reliable and precise way under the control of brain stem nuclei, which act as central pattern generators (Travers et al., 1997). To understand which areas of the dorsal cortex are involved, we first performed awake optogenetic motor mapping experiments. In a transgenic mouse line expressing channelrhodopsin-2 in excitatory cortical neurons (Arenkiel et al., 2007), different locations of the dorsal cortex were focally stimulated using a blue laser while facial movements were filmed with a high-speed camera to track C2 whisker movement and jaw opening (Figures 1A and S1A; STAR Methods). Whisker protraction was evoked by stimulation of a cortical region (wM1) centered at 1.4 ± 0.2 mm lateral and 1.9 ± 0.3 mm anterior to bregma (n = 6 mice) (Table S1A) (Auffret et al., 2018). In the same mice, we found that jaw opening was evoked by stimulation of a cortical region (tjM1) centered at 2.6 ± 0.1 mm lateral and 1.8 ± 0.5 mm anterior to bregma. These maps thus show distinct cortical epicenters for initiation of whisker and jaw movements.

We next investigated whether we could detect sensory-evoked activity in frontal cortex, as a way to localize primary motor cortex. Wide-field calcium imaging of the left hemisphere of the dorsal cortex was performed through the intact skull of transgenic mice expressing the genetically encoded calcium indicator GCaMP6f (Dana et al., 2014) (Figure 1B). Mice were lightly anesthetized with isoflurane and vibrotactile stimuli were applied consecutively to the C2 whisker and the tip of the tongue. Mechanical stimulation of the tongue was accompanied by jaw vibrations. Early responses (0–100 ms after stimulus onset) were evoked in sensory cortex, centered at 3.4 ± 0.3 mm lateral and 1.4 ± 0.2 mm posterior to bregma for C2 whisker deflection (wS1) and at 3.8 ± 0.3 mm lateral and 0.0 ± 0.3 mm posterior to bregma for tongue-jaw stimulation (tjS1) (n = 5 mice) (Figure S1B; Table S1B). In a later time window (100–200 ms after stimulus onset), a distinct second activity spot appeared in frontal cortex, centered at 1.3 ± 0.4 mm lateral and 1.5 ± 0.3 mm anterior to bregma for C2 whisker deflection (wM1) and at 2.4 ± 0.6 mm lateral and 1.8 ± 0.1 mm anterior to bregma for tongue-jaw stimulation (tjM1).

To investigate anatomical connectivity from tjS1 to tjM1, we expressed a red fluorescent protein through injection of an adeno-associated viral vector into tjS1 mapped using wide-field calcium imaging. Serial two-photon tomographic imaging of the whole brain revealed a strong axonal innervation in the frontal portion of the cortex (tjM1) centered at 2.1 ± 0.1 mm lateral and 1.5 ± 0.2 mm anterior to bregma (n = 5 mice) (Figures 1C and S1C; Table S1C; Video S1).

Video S1. Image Stack Using Serial Two-Photon Tomography, Related to Figure 1 A movie of serial coronal sections from injection site (tjS1) to anterior slices where axons innervate the motor cortex (tjM1). The data are downsampled by a factor of 6 for X and Y, and by a factor of 10 for Z.

Three independent methods therefore localize tjM1 within a region 2.1–2.6 mm lateral and 1.5–1.8 mm anterior to bregma (Table S1).

### Different Roles of wS1 and tjM1 during Goal-Directed Behaviors

**Figure 2:**
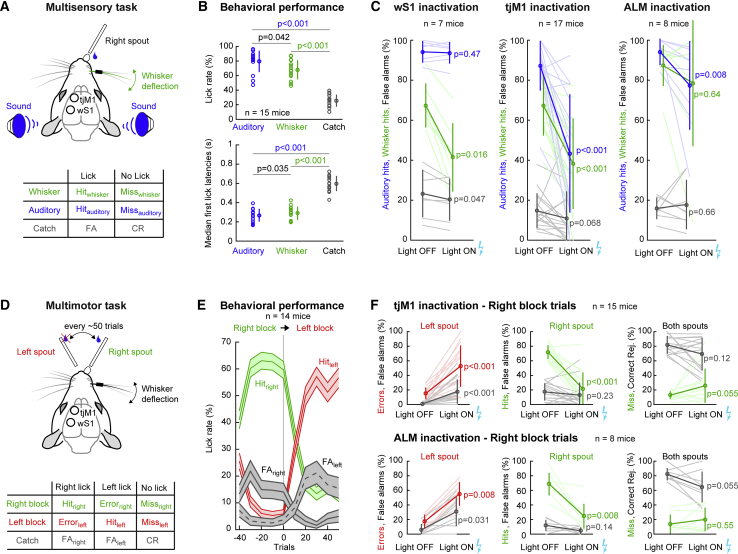
Different Roles of tjM1 and wS1 in Sensorimotor Behaviors (A) In a multisensory detection task, mice were rewarded by licking a spout within a 1.5 s time window following a brief whisker deflection (1 ms) or a short auditory tone (10 ms). Catch trials with no stimulus and no reward were interleaved. Table below describes the categories of possible trial types and behavioral outcomes. (B) Lick probability (above) and median first lick latency (below) for auditory (blue), whisker (green), and catch (gray) trials for expert mice (n = 15 mice). (C) Lick probability for the auditory (blue), whisker (green), and catch (gray) trials during optogenetic inactivation of wS1 (n = 7 mice), tjM1 (n = 17 mice), and ALM (n = 8 mice). (D) In a multimotor detection task, mice were trained to lick a spout on the right or left in response to whisker deflection. Every ∼50 trials the reward location was changed between right and left spouts. (E) Lick probability for right spout (green) and left spout (red) aligned to the spout switch event (n = 14 mice). The lick probability for the catch trials is shown in gray (right spout, solid; left spout, dashed). (F) tjM1 and ALM inactivation during the multimotor task. Upper graphs show the effect of tjM1 inactivation (n = 15 mice) on left and right spout lick probability during right block trials, as well as miss rates. Lower graphs show the same, but during ALM inactivation (n = 8 mice). Data are represented as mean ± SD, except for 95% confidence intervals in (E). Wilcoxon signed-rank test is shown in (B), (C), and (F). See also Figure S2.

To study the role of wS1 and tjM1 during perceptual decision-making, we trained mice to lick a reward spout in response to randomly interleaved presentations of a brief whisker stimulus or a brief auditory stimulus (Figure 2A). Mice learned this multisensory detection task within a week (auditory hit rate: 79% ± 15%; whisker hit rate: 69% ± 13%; false alarm rate: 26% ± 8%; p < 0.001 for both auditory and whisker hit rates compared to false alarm rate; Wilcoxon signed-rank tests, n = 15 mice, quantified during two-photon imaging sessions, see below) (Figures 2B, S2A, and S2B). First lick latencies were similar in whisker and auditory hit trials (269 ± 67 ms for auditory hit; 290 ± 68 ms for whisker hit) but different for false alarm trials (599 ± 79 ms; p < 0.001; Wilcoxon signed-rank tests) (Figures 2B and S2B). These observations demonstrate a conditioned motor action (licking) upon sensory stimulation (whisker or auditory).

In additional mice, we carried out inactivation experiments to investigate the causal roles of tjM1 and wS1 during task execution. Optogenetic inactivation was performed by locally and transiently stimulating inhibitory neurons expressing channelrhodopsin-2 in a transgenic mouse line (Zhao et al., 2011). Blue light pulses were delivered through the transparent skull in a subset of randomly interleaved trials to silence either tjM1 or wS1. Inactivation of wS1 led to a specific reduction of licking in response to whisker deflections but not to auditory tones (whisker hit rate: 67% ± 11% to 41% ± 17%, p = 0.016; auditory hit rate: 94% ± 5% to 94% ± 5%, p = 0.47; Wilcoxon signed-rank tests, n = 7 mice) (Figure 2C, left). In contrast, inactivation of left tjM1 led to reduced licking on a spout positioned on the right side (contralateral to the inactivation side) in response to both whisker and auditory stimuli (whisker hit rate: 67% ± 15% to 38% ± 23%, p < 0.001; auditory hit rate: 87% ± 12% to 43% ± 30%, p < 0.001; Wilcoxon signed-rank tests, n = 17 mice) (Figure 2C, middle). Similar results were obtained in a set of experiments in which the GABA receptor agonist muscimol was infused in wS1 or tjM1 (Figure S2C).

Recently, the anterior lateral motor (ALM) cortex was shown to be involved in motor planning during a delayed whisker-dependent licking task (Guo et al., 2014, Svoboda and Li, 2018). We therefore tested the role of ALM in our multisensory task. Optogenetic inactivation of ALM, located at 1.5 mm lateral and 2.5 mm anterior to bregma, only had a small effect upon task performance (whisker hit rate: 87% ± 10% to 79% ± 32%, p = 0.64; auditory hit rate: 94% ± 6% to 77% ± 22%, p = 0.008; Wilcoxon signed-rank tests, n = 8 mice) (Figure 2C, right). The reduction in hit rate was significantly larger for inactivation of tjM1 in comparison to ALM (whisker p = 0.029; auditory p = 0.025; Wilcoxon rank-sum tests). Consistent with previous results (Le Merre et al., 2018), we found that inactivation of wM1 did not change hit rates (whisker hit rate: 89% ± 7% to 95% ± 4%, p = 0.11; auditory hit rate: 95% ± 8% to 95% ± 9%, p = 0.63; Wilcoxon signed-rank tests, n = 8 mice).

The same mice were next trained to lick a spout either on the right or the left in response to a brief whisker deflection. In this multimotor task, the rewarded direction of licking was kept constant during a block of trials (left block or right block trials), which switched every ∼50 trials (Figure 2D). Mice successfully changed the side of licking within 10–20 trials from the switch time (Figures 2E, S2D, and S2E). During optogenetic inactivation of left tjM1 in right block trials, licking was reduced for the right spout and increased for the left spout (hits right spout: 72% ± 10% to 22% ± 23%, p < 0.001; errors left spout: 15% ± 8% to 53% ± 28%, p < 0.001; misses: 13% ± 5% to 26% ± 23%, p = 0.055; Wilcoxon signed-rank tests, n = 15 mice) (Figure 2F, top). In contrast to the multisensory task, in the multimotor task, inactivation of left ALM had a comparable effect to inactivation of tjM1 (hits right spout: 69% ± 15% to 24% ± 17%, p = 0.008; errors left spout: 17% ± 9% to 55% ± 16%, p = 0.008; misses: 14% ± 13% to 20% ± 16%, p = 0.55; Wilcoxon signed-rank tests, n = 8 mice) (Figure 2F, bottom). Optogenetic inactivation of tjM1 and ALM also affected performance in left block trials (Figure S2F). Inactivation of wM1 in right block trials did not affect hit rates (hits right spout: 66% ± 14% to 65% ± 19%, p = 0.74; errors left spout: 17% ± 14% to 26% ± 19%, p = 0.08; Wilcoxon signed-rank tests, n = 8 mice).

Although it is important to note limitations in optogenetic inactivation experiments including their impact upon downstream brain areas and possible homeostatic compensations, our results suggest that wS1 is necessary for detection of whisker stimuli and that tjM1 is important for contralateral licking regardless of the sensory modality used to trigger this motor output.

### Neuronal Correlates of Multisensory and Multimotor Decision Making

**Figure 3:**
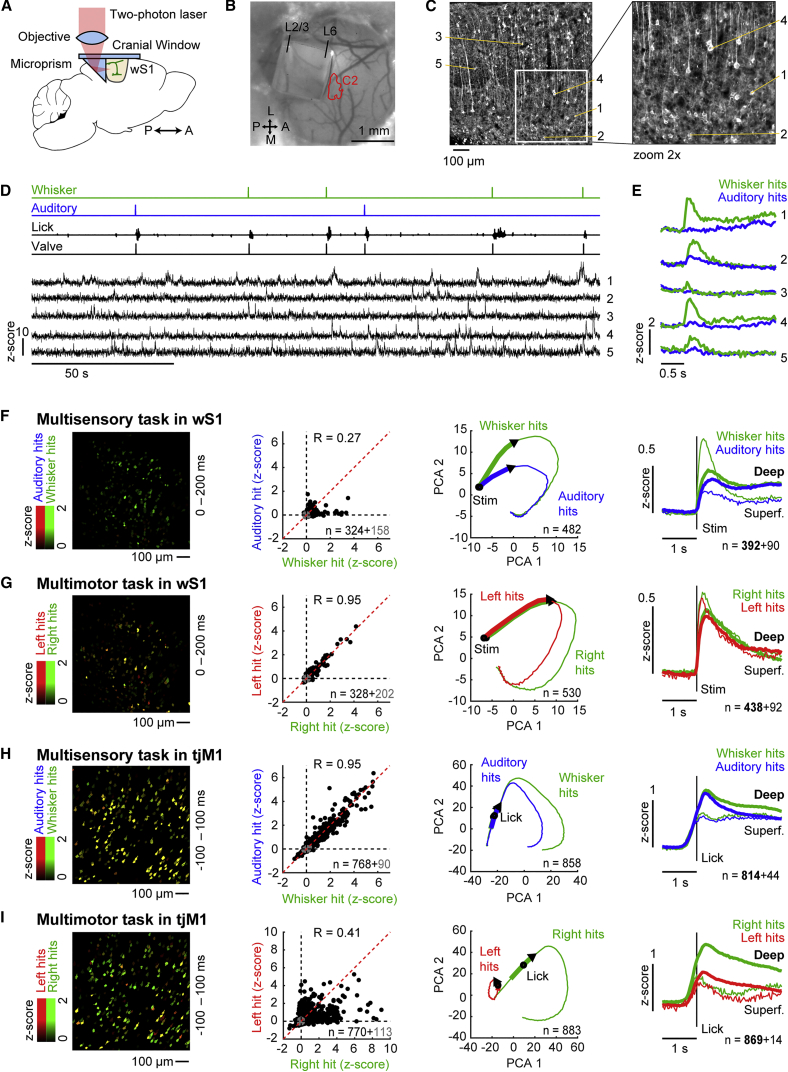
Neuronal Correlates of Multisensory and Multimotor Decision Making (A) A microprism assembly was inserted into the cortex for chronic two-photon calcium imaging across layers. (B) Top view of an implanted microprism assembly targeted to wS1. The red contour indicates the peak of the intrinsic optical signal evoked by C2 whisker stimulation. (C) Laminar view of cortical neurons in a Thy1-GCaMP6f mouse imaged through the microprism assembly shown in (B). (D) Traces of behavioral variables and calcium signals from five example neurons (labeled in C) during the multisensory detection task. (E) Average *Z* score calcium responses aligned to stimulus onset. (F) Left: example field of view during the multisensory task in wS1. Red image channel: average stimulus-triggered responses (0–200 ms) for auditory hit trials. Green image channel: the same but for whisker hit trials. Middle left: scatterplot over all neurons comparing average response during whisker and auditory hit trials (0–200 ms post stimulus). Black points indicate significantly modulated neurons. Gray points represent neurons that did not show any significant modulation. R indicates the Pearson correlation coefficient. Middle right: PCA of the time-varying neuronal population vector during whisker and auditory hit trials. Thick lines indicate the first 200 ms after stimulus onset. Arrow heads show direction of the trajectories. Right: grand average whisker and auditory *Z* score evoked responses in hit trials for deep (thick) and superficial (thin) neurons. (G) Left: example field of view during the multimotor task in wS1 (left). Red image channel: average stimulus-triggered responses (0–200 ms) for left hit trials. Green image channel: the same but for right hit trials. Middle left: scatterplot over all neurons comparing average response during left and right hit trials (0–200 ms post stimulus). Black points indicate significantly modulated neurons. Gray points represent neurons that did not show any significant modulation. R indicates the Pearson correlation coefficient. Middle right: PCA of the time-varying neuronal population vector during left and right hit trials. Thick lines indicate the first 200 ms after stimulus onset. Arrows show direction of the trajectories. Right: grand average left and right *Z* score evoked responses in hit trials for deep (thick) and superficial (thin) neurons. (H and I) Same as (F) and (G) but for two-photon imaging in tjM1. Average responses were calculated in the time window −100–100 ms relative to first lick time. Pearson correlation coefficients in (F)–(I): p < 0.001. See also Figure S3.

We next characterized neuronal responses during task performance by performing two-photon calcium imaging in Thy1-GCaMP6f mice (Dana et al., 2014). To chronically monitor the activity of neurons in superficial and deep cortical layers (>500 μm), we used a cranial window assembly comprising a microprism (Andermann et al., 2013, Bird and Gu, 2003, Murayama et al., 2009, Takahashi et al., 2016) that was inserted in either wS1 or tjM1 (Figures 3A–3C and S3A). Regions of interest were automatically detected and further used to extract neuronal calcium signals (Figure S3A) (Pachitariu et al., 2017). Aligning calcium responses to different sensory or motor events allowed us to assess whether these events significantly modulated single neurons during task performance (Figures 3D and 3E).

In wS1, calcium responses were different comparing whisker and auditory hit trials in the early phase (0–200 ms following stimulus onset) (Figure 3F). Across all task-modulated neurons, we found that whisker hit trials evoked stronger responses compared to auditory hit trials (average *Z* score response for whisker hits 0.25 ± 0.49 versus auditory hits 0.11 ± 0.21; p < 0.001, Wilcoxon signed-rank test; n = 324 significantly modulated neurons out of 482 neurons from 13 fields of view in 7 mice) (Frostig et al., 2008, Iurilli et al., 2012, Maruyama and Komai, 2018). For this early response, similar results were found in miss trials (Figure S3). We used principal-component analysis (PCA) to visualize the time-varying neuronal network trajectory. Projected onto the two-dimensional space defined by the first two PCA components, activity in wS1 followed a different trajectory in the early phase of whisker hit trials compared to auditory hit trials. At later times, the population activity became more similar between the two conditions. Interestingly, the differential response to whisker versus auditory stimuli was more prominent in superficial neurons compared to deep neurons (average *Z* score response for whisker hits superficial 0.51 ± 0.75 versus auditory hits superficial 0.09 ± 0.17, p < 0.001, n = 67; whisker hits deep 0.19 ± 0.37 versus auditory hits deep 0.12 ± 0.23, p = 0.03, n = 257; Wilcoxon signed-rank tests).

In the multimotor task, calcium responses in wS1 during the early phase after stimulus onset were similar comparing right versus left hit trials (Figure 3G) (average *Z* score response for right hits 0.32 ± 0.55 versus left hits 0.32 ± 0.56; p = 0.61, Wilcoxon signed-rank test; n = 328 significantly modulated neurons out of 530 neurons from 14 fields of view in 8 mice). At later times, we observed a separation of network trajectories between left and right lick trials. Aligning the analyses on the first lick timing for each trial gave similar results (Figures S3B and S3C).

Calcium responses in tjM1 were similar between whisker hit trials and auditory hit trials (averaged ± 100 ms around tongue-spout contact time) (Figure 3H). We found a strong correlation between responses in auditory hit trials and whisker hit trials for individual neurons, and little divergence in the first two PCA dimensions of the population activity trajectories comparing auditory and whisker hit trials (average *Z* score response for whisker hits 0.66 ± 1.01 versus auditory hits 0.67 ± 1.00; p = 0.15, Wilcoxon signed-rank test; n = 768 significantly modulated neurons out of 858 neurons from 12 fields of view in 7 mice).

In contrast, when comparing left versus right hit trials, the calcium activity in tjM1 was different for left and right licks (Figure 3I). Correlation between single-neuron responses in these two conditions was weak, and the grand average response was higher for right hits compared to left hits (average *Z* score response for right hits 0.88 ± 1.31 versus left hits 0.45 ± 0.76; p < 0.001, Wilcoxon signed-rank test; n = 770 significantly modulated neurons out of 883 neurons from 11 fields of view in 6 mice). Similar results were obtained by aligning the data to the stimulus onset (Figures S3D and S3E).

Hence, neuronal responses in wS1 displayed a sensory signature biased toward whisker hit trials (especially for superficial neurons), whereas tjM1 neurons differentiate better between motor actions with a bias toward right (contralateral) licks.

### Decoding of Lick Directions and Small Anticipatory Movements

**Figure 4:**
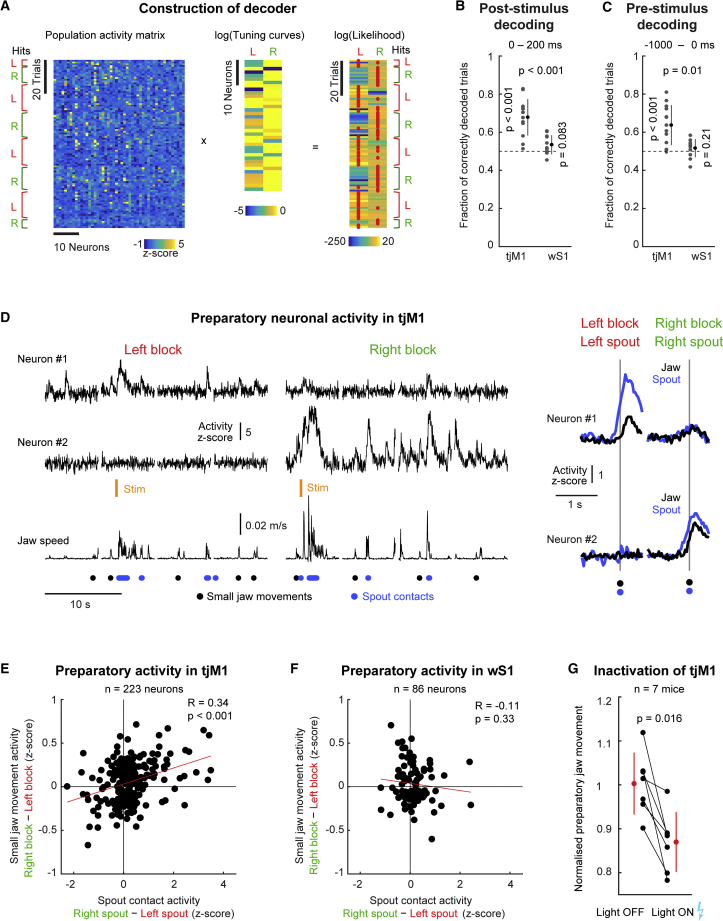
Decoding of Lick Direction and Small Anticipatory Movements (A) Computation of the maximum log-likelihood for decoding motor output. Left: a population activity matrix of error and hit trials for a tjM1 example session. Middle: the log-tuning curves for left and right licks are shown for all cells in one field of view. Right: matrix multiplication of the population activity matrix with the log-tuning curves (plus the same bias correction for each row, data not shown; see STAR Methods for details) leads to the log-likelihood for left and right lick in each trial. Choosing the side (L or R) with the highest value for each row (trial) gives a prediction of lick direction (red dots). (B) Decoder performance for a post-stimulus (0–200 ms) time window in tjM1 (n = 13 fields of view in 5 mice) and wS1 (n = 11 fields of view in 6 mice). Each gray point indicates the decoder performance of a single field of view and session. Black points represent grand averages. Dashed line shows chance level. (C) Same as (B) but for pre-stimulus (−1,000–0 ms) time window. (D) Left: calcium activity of two tjM1 example neurons during left and right block trials and corresponding jaw movements extracted from facial filming. Black dots, onset of isolated small jaw movements from the facial filming. Blue dots, spout contacts. Orange line, whisker stimulus timing. Right: average response of the neurons aligned to spout contact (blue) or on isolated small jaw movements (black). (E) Population analysis of (D) for tjM1. Differences between right and left spout contact responses were correlated to differences between right and left block responses during small jaw movements. (F) Same as (E) but for wS1. (G) Analysis of small jaw movements in catch trials during tjM1 optogenetic inactivation experiments. Each black point corresponds to the average ratio of jaw movements in a baseline window to jaw movements in the response window with light OFF or light ON (n = 7 mice). Data are represented as mean ± SD. Wilcoxon rank-sum test: tjM1 versus wS1 in (B) and (C); Wilcoxon signed-rank test: tjM1 and wS1 against chance in (B) and (C). Pearson correlation coefficient is shown in (E) and (F). Wilcoxon signed-rank test is shown in (G). See also Figure S4.

To further investigate the role of tjM1 in coding lick direction, we made use of a Bayesian approach based on the idea of a probabilistic population code (Jazayeri and Movshon, 2006, Ma et al., 2006). For each field of view, we computed the tuning curves for spontaneous left and right licks (Figure 4A). By summing the product of the log-tuning curves and the population activity vector for each trial, we obtained the log-likelihood for left and right lick in the corresponding trial. The maximum log-likelihood can then be used as a decoder of the motor action. Decoder performance was measured by the fraction of trials where the lick direction was correctly decoded from neuronal activity.

Running this decoder on a time window of 0–200 ms following stimulus onset in trials with evoked licking (hit and error trials) indicates that tjM1, but not wS1, neuronal activity can be used to decode lick direction well above chance level (p < 0.001 for tjM1 versus p = 0.083 for wS1 against chance, Wilcoxon signed-rank tests; tjM1 versus wS1: p < 0.001, Wilcoxon rank-sum test; for tjM1 n = 13 fields of view from 5 mice; for wS1 n = 11 fields of view from 6 mice) (Figures 4B and S4A). Surprisingly, by applying the decoder on a window preceding the stimulus onset (−1,000–0 ms), we also found a decoding performance above chance level for tjM1 but not for wS1 (p = 0.008 for tjM1 versus p = 0.21 for wS1 against chance, Wilcoxon signed-rank tests; tjM1 versus wS1: p < 0.001, Wilcoxon rank-sum test; for tjM1 n = 13 fields of view from 5 mice; for wS1 n = 11 fields of view from 6 mice) (Figures 4C and S4B). During this pre-stimulus time window, no tongue contacts on either spout were detected. To better understand the nature of this anticipatory neuronal activity, we used the high-speed behavioral filming data to assess whether the neuronal activity was correlated to any overt behavior. We found small movements of the jaw during the pre-stimulus period in both left and right blocks of trials (Figures 4D, S4C, and S4D). When we aligned the neuronal calcium activity to these small jaw movements in the left and right block, we found that these movements correlated with neuronal calcium responses that were selective to the trial block. An example left lick tuned neuron showed responses during small jaw movements in the left block but not in the right block (Figure 4D, cell #1). Similarly, an example right lick tuned cell showed more response accompanying the small jaw movements during the right block compared to the left block (Figure 4D, cell #2). For all neurons with significant responses to spontaneous licks, we computed the right-left tuning and correlated this with the right-left block difference in activity during small preparatory jaw movements. We found a significant correlation in tjM1 (R = 0.34, p < 0.001 for tjM1, n = 223 neurons from 13 fields of view from 5 mice) (Figure 4E), but not in wS1 (R = −0.11, p = 0.33 for wS1, n = 86 neurons from 10 fields of view from 7 mice) (Figure 4F).

To assess the causal relationship between activity in tjM1 and these small preparatory jaw movements, we analyzed correct rejection catch trials (trials in absence of stimulus and spout contact) in the optogenetic inactivation experiments (Figure 2). In inactivation trials, we quantified change in jaw movement by comparing the SD of the filmed jaw position before and during light delivery. This was then compared to trials where no light was delivered (Figure S4E). We observed a significant reduction of small jaw movements during optogenetic inactivation of tjM1 but not wS1 (ratio of jaw movement during tjM1 inactivation relative to baseline 0.87 ± 0.07, p = 0.016; during wS1 inactivation 0.97 ± 0.16, p = 0.30; Wilcoxon signed-rank tests, n = 7 mice) (Figure 4G).

Lick direction could therefore be decoded from neuronal activity in tjM1 but not from wS1. Preparatory neuronal activity preceding stimulus delivery in each block was directly related to the upcoming lick direction and correlated with small jaw movements of the corresponding block. Inactivation experiments indicated a causal involvement of tjM1 in driving small preparatory movements.

## Discussion

In our study, we combined anatomical and functional methods to define a tongue and jaw related primary motor region of mouse frontal cortex (tjM1) centered around 2.1–2.6 mm lateral and 1.5–1.8 mm anterior of bregma (Figure 1). Neurons in tjM1 may participate in licking motor control through different pathways, likely including direct projections to premotor neurons located in the brainstem reticular formation (Travers et al., 1997). We found that tjM1 neurons encode licking direction and not the sensory stimulus used to initiate licking (Figure 3). Optogenetic as well as pharmacological inactivation experiments showed that tjM1 neuronal activity is necessary for contralateral licking in our task (Figures 2 and S2).

Neuronal activity in tjM1 carried sufficient information to decode lick direction not only during the response window but also before the stimulus had been delivered (Figure 4). In this pre-stimulus time window, we observed small jaw movements, which were correlated with preparatory neuronal activity. Inactivation experiments indicated a causal involvement of tjM1 in driving these preparatory movements. The direction-specific preparatory activity in tjM1 might be related to anticipatory neuronal activity (or working memory) in delay tasks (Chen et al., 2017, Guo et al., 2014, Li et al., 2016). Our results raise the possibility that preparatory neuronal activity, and perhaps working memory, might in general be accompanied by measurable motor output.

The first wave of neuronal activity in wS1 after stimulus time encoded whisker sensation and not directional motor output. Optogenetic (Figure 2) and pharmacological (Figure S2) inactivation experiments revealed that primary sensory whisker cortex played a key role in the whisker detection task in which mice were trained in this study. Inactivating wS1 specifically impaired detection of whisker stimuli without affecting licking motor control, since auditory detection performance was unaltered. These data are in good agreement with previous pharmacological and optogenetic inactivation experiments testing the role of wS1 in whisker detection tasks (Le Merre et al., 2018, Miyashita and Feldman, 2013, Sachidhanandam et al., 2013, Yang et al., 2016).

In future experiments, it will be of great interest to understand how whisker sensory information is routed from wS1 to tjM1 to initiate licking after task learning. Specific subsets of wS1 neurons project to different targets and previous work has suggested the importance of signaling from wS1 to wS2 (Chen et al., 2013, Kwon et al., 2016, Yamashita and Petersen, 2016) and to striatum (Sippy et al., 2015). Inactivation of wS2, but not wM1, causes a decrease in hit rate (Le Merre et al., 2018), suggesting the importance of signaling via wS2 in whisker detection tasks. However, neither wS2 nor striatum innervate tjM1, and so further intermediate brain regions must participate. Neurons in tjM1 likely receive input from many sources and future experiments must uncover the relevant signaling pathways. Interestingly, the licking premotor area ALM appeared to contribute mainly in left versus right choice tasks (Figure 2F). Premotor areas, such as the ALM, might thus be recruited in more complex decision making, and could provide an important input to primary motor areas, such as tjM1, contributing to task execution.

## STAR★Methods

### Key Resources Table

**Table:** 

REAGENT or RESOURCE	SOURCE	IDENTIFIER
**Chemicals, Peptides, and Recombinant Proteins**		

Muscimol [2763-96-4]	Biotrend USA	Cat# BN0352

**Experimental Models: Bacterial and Viral Strains**		

AAV1.hSyn.TurboRFP.WRPE.rBG	UPenn Vector Core	AV-1-PV2642

**Experimental Models: Organisms/Strains**		

Mice Thy1-GCaMP6f	The Jackson Laboratory	Jax025393
Mice Thy1-ChR2	The Jackson Laboratory	Jax07612
Mice VGAT-ChR2	The Jackson Laboratory	Jax014548

**Deposited Data**		

Dataset and MATLAB analysis code	this paper	https://doi.org/10.5281/zenodo.3271408

**Software and Algorithms**		

MATLAB > R2014b	MathWorks	https://www.mathworks.com/
Labview	National Instruments	http://www.ni.com/en-us.html
Suite2p	https://github.com/cortex-lab/Suite2P	Pachitariu et al., 2017
NoRMCorre	https://github.com/flatironinstitute/NoRMCorre	Pnevmatikakis and Giovannucci, 2017
BakingTray	https://github.com/SainsburyWellcomeCentre/BakingTray	Han et al., 2018
StitchIt	https://github.com/SainsburyWellcomeCentre/StitchIt	Han et al., 2018
MaSIV	https://github.com/SainsburyWellcomeCentre/masiv	Han et al., 2018

### Lead Contact and Materials Availability

Further information and requests for resources and reagents should be directed to and will be fulfilled by the Lead Contact, Carl Petersen (carl.petersen@epfl.ch). This study did not generate new unique reagents.

### Experimental Model and Subject Details

All procedures were approved by Swiss Federal Veterinary Office (License number VD1628) and were conducted in accordance with the Swiss guidelines for the use of research animals. Thy1-GCaMP6f (C57BL/6J-Tg(Thy1-GCaMP6f)GP5.17Dkim/J, JAX mouse number 025393), Thy1-ChR2 (B6.Cg-Tg(Thy1-COP4/EYFP)18Gfng/J, JAX mouse number 07612), and VGAT-ChR2 (B6.Cg-Tg(Slc32a1-COP4^∗^H134R/EYFP)8Gfng/J, JAX mouse number 014548) transgenic mouse lines were used. Both male and female mice were used, and the mice were at least 6 weeks of age at the time of head-post implantation (see below).

### Method Details

#### Experimental design

This study did not involve randomization or blinding. We did not estimate sample-size before carrying out the study. No data or subjects were excluded from the analysis.

#### Surgery

Mice were anesthetized with 2 – 4% isoflurane (Attane, USA) in pure oxygen, or with a mixture of ketamine and xylazine injected intraperitoneally (ketamine: 125 mg/kg, xylazine: 10 mg/kg). Body temperature was monitored and kept at 37°C throughout the surgery with the help of a body temperature controlled heating pad. An eye cream (Viscotears, Alcon, USA; VITA-POS, Pharma Medica AG, Switzerland) was applied over the eyes to prevent them from drying. Carprofen was injected intraperitoneally or subcutaneously (100 μl at 0.5 mg/ml or 100 μl at 1.5 mg/ml) for analgesia. As local analgesic, a mix of lidocaine and bupivacaine was injected below the scalp before any surgical intervention. As a general analgesic treatment, ibuprofen (Algifor Dolo Junior, VERFORA SA, Switzerland) was given in the drinking water for three days after surgery. A povidone-iodine solution (Betadine, Mundipharma Medical Company, Bermuda) was used for skin disinfection before surgery. To access the dorsal cortex, a part of the scalp was removed with surgical scissors. The remaining connective tissue was removed with a scalpel blade. After disinfecting the skull with Betadine and rinsing it with Ringer solution, it was dried with cotton buds. A layer of super glue was then applied and a custom-made head fixation implant was glued (Loctite super glue 401, Henkel, Germany) to the right hemisphere of the skull. The head implant was further secured with self-curing denture acrylic (Paladur, Kulzer, Germany; Ortho-Jet, LANG, USA). Particular care was taken to ensure that the left hemisphere of the dorsal cortex was free of denture acrylic and only covered by a thin layer of super glue for optical access. This transparent skull preparation was used to perform wide field imaging, optogenetic activation, and inactivation experiments. After three days of recovery, intrinsic optical signal (IOS) imaging was performed on the left hemisphere as previously described (Sachidhanandam et al., 2013). A piezoelectric actuator was used to repeatedly stimulate either the tongue or the right C2 whisker. Increase in absorption of red light (625 nm) upon tactile stimulation indicated the functional location of the tongue-jaw and the C2 whisker in the somatosensory cortex, respectively. To perform two-photon calcium imaging, a craniotomy of about 3 mm was made either above C2 whisker primary somatosensory cortex (based on the IOS) or above tongue-jaw primary motor cortex (based stereotaxic coordinates: 2 mm anterior and 2 – 2.3 mm lateral relative to bregma). For superficial imaging a window assembly of two 3 mm and one 5 mm round coverslips (CS-3R and CS-5R, Warner Instruments, USA) were bound by a light curing adhesive (NOA61, Thorlabs, USA) and fitted into the craniotomy. To access deeper layers of the cortex, a microprism window assembly (Andermann et al., 2013) was inserted either in C2 barrel of wS1 along the medial-lateral axis or in tjM1 along the anterior-posterior axis (Figures 3A–3C). The microprism window assembly consisted of a prism coated with aluminum (MPCH-1.0 for wS1 and MPCH-1.5 for tjM1, Tower Optical Corporation, USA), two 3 mm and 5 mm window glued together with the light curing adhesive. The window assemblies were fixed with super glue and denture acrylic to the skull. In experiments where microprisms were inserted in the cortex, at least 5 weeks of recovery were given for cortical tissue to stabilize before any imaging was performed whereas one to two weeks were sufficient for conventional window surgeries.

#### Behavioral training and monitoring

Mice were subjected to a water restriction schedule. The multisensory detection paradigm consisted of single whisker deflections (small iron particle attached to the C2 whisker was deflected by a rapid change of a magnetic field (Sachidhanandam et al., 2013) with an effective force duration of ∼1 ms) and brief auditory tones (10 ms long 10 kHz pure tone of 3 dB added on top of the continuous masking white noise at 80 dB). Training started with an associative learning phase where sensory stimuli (whisker and auditory stimulus) were followed by an automated delivery of 4 μl of water. As soon as the mouse engaged in licking from the spout, normal trials where reward was delivered only upon licking were interleaved. After one to two training sessions, associative trials were completely omitted. Behavior was analyzed with MATLAB (MathWorks, USA). Associative trials were not included in the analysis. To measure spontaneous licking rate, catch trials in which no stimulus was delivered were interleaved with other trials. The average interstimulus interval was 12 ± 1 s (including catch trials with a virtual stimulus time). The stimulus delivery only occurred after a quiet period during which mice did not lick the spout for 3 s. The window during which mice could be rewarded was 1.5 s long and started at the stimulus onset. To measure small facial movements, behavioral filming of the silhouette of the mouse’s face was performed with a high-speed camera (CL 600 X 2/M, Optronis, Germany) running at a frame rate of 200 Hz, an exposure time of 4 ms and a resolution of 128x128 pixels. In a subset of experiments, filming from below the mouse was performed at frame rate of 100 Hz with an exposure time of 4 ms and a resolution of 192x192 pixels. For the multimotor detection paradigm, mice were first trained on the standard multisensory detection task with the spout placed on the right side. Once they became expert at this task, they were trained to lick for blocks of about 50 trials on the right or left spout upon a brief whisker deflection. After 50 trials the reward location was changed to the spout on the opposite side. To indicate the current reward location associative trials were interleaved. The control and acquisition hardware (NI PXI-6259, NI PXI-6711, NI PCI-6221, National Instruments, USA) for the behavior setup PC and filming PC were controlled by custom-made software written in Labview code (National Instruments, USA).

#### Optogenetics

For optogenetic inactivation experiments, a 200 μm (BFH37-200, Thorlabs, USA) or 400 μm diameter fiber (BFH37-400, Thorlabs, USA) attached to a 470 nm high power LED (M470F3, Thorlabs, USA) or laser (MBL-F-473/200mW, Changchun New Industries Optoelectronics Technology, China) was used to selectively activate in a subset of randomly interleaved trials (about 1/3) GABAergic neurons in wS1, tjM1 or ALM (based on IOS maps or coordinates). The light stimulus consisted of a 100 Hz pulse train with duty cycle of 50%. The average power output at the fiber end was measured within a range of 4.5 – 20 mW. For awake optogenetic stimulation mapping of the cortex, we used a custom-built setup to steer a blue laser beam (S1FC473MM, Thorlabs, USA) at different locations of the dorsal cortex (Auffret et al., 2018). By introducing a pair of scan and tube lenses (AC508-100-A, Thorlabs, USA), the whole area of the dorsal cortex was made accessible. Optogenetic stimulation (1 mW peak power, ∼0.4 mm beam diameter, 50 Hz sinusoidal wave, 500 ms duration) was pseudo-randomly delivered to each location of the dorsal cortex following a grid pattern with 0.5 mm spacing. Movements of the right C2 whisker and the jaw were measured under blue light illumination by a high-speed camera (CL 600 X 2/M, Optronis, Germany; 500 Hz frame rate, 0.5 or 1 ms exposure time, and 512x512 pixels resolution). Movements were tracked by using a custom-made MATLAB code. Briefly, arc regions-of-interest were defined around the basal points for both the whisker and jaw. Crossing points on these arcs were detected for the whisker (the pixels with the smallest intensity) and the jaw (pixels with the largest slope in intensity). A vector was then defined for each pair of basal point and the cross point, and the absolute angle was calculated for each vector. Trials with optogenetic stimulations were included in the analysis only when the standard deviation of movement angles during the pre-stimulation periods (100 ms for whisker, 250 ms for jaw) did not exceed a threshold criterion (2 deg for whisker, 0.75 deg for jaw). For each stimulation trial, optogenetically-evoked movements were calculated as the difference in mean angles between a 4-ms period immediately before the stimulation and the first 200-ms period during the stimulation. Positive and negative values were defined as protraction and retraction for the whisker, and opening and closure for the jaw, respectively. For each mouse, optogenetically-evoked movements were mapped on the stimulated cortical location and were further averaged across all stimulation trials. For the primary motor cortices in each map, center coordinates were determined as the weighted centroid of the pixels in which values exceed 50% of the maximum. Coordinates of primary sensory cortices were also determined in each mouse as the centroid of the sensory responses in intrinsic optical signal imaging. The maps of optogenetically-evoked responses were averaged across six mice by aligning to C2 whisker signal because of its well-defined localization across mice.

#### Wide-field imaging

Mice were lightly anesthetized (1 – 1.2% isoflurane in 100% oxygen) and mounted with an angle of about 25° rotation along the anterior-posterior axis. Either the C2 whisker or the tongue was stimulated using a piezoelectric actuator following a 25 Hz sine-wave displacement for at least 200 ms duration. To access the tongue for vibriotactile stimulations, the jaw was opened and the tongue was pulled gently with forceps and dried with cotton buds. The GCaMP6f calcium indicator was excited with blue light at 485 nm (halogen lamp, TH4-200 and U-LH100-3, Olympus, Japan; 485/20 BrightLine HC, Semrock, USA) and emission light was detected through a green band pass filter (525/50 BrightLine HC, Semrock, USA). A dichroic mirror (Beamsplitter T 495 LPXR, Chroma Technology Corp, USA) was used to separate excitation and emission light. The left dorsal hemisphere of the cortex was projected on a CMOS chip by using a face-to-face tandem objective (Nikkor 50mm f/1.2, Nikon, Japan; 50 mm video lens, Navitar, USA). Images were acquired at a resolution of 100x100 pixels (100 μm / pixel) and a frame rate of 100 Hz with a 12 bit camera (MiCAM Ultima, Scimedia, USA). Stimuli and hardware synchronization was done with MATLAB and a National Instrument card (NI PCIe-6342) running on a PC. To collect an anatomical reference image for each imaging session, the top of the transparent skull was illuminated with a fiber (M71L02 - Ø1000 μm, 0.48 NA, SMA-SMA Fiber Patch Cable, Thorlabs, USA) coupled to a green LED (530 nm, M530F2, Thorlabs, USA). During image acquisition, a single trial consisted of a 2 s-long pre-stimulus period followed by 3.12 s post stimulus. For each stimulus condition, 50 repetitions were acquired. The first step of image processing was to average those trials for each stimulus condition to create an average movie F, where F(i,j,t) indicates the fluorescence of single pixel (ith row, jth line) measured at time point t. A baseline image F_0_(i,j) was then calculated by averaging the last 5 frames prior to the stimulus onset (−50 – 0 ms). Finally, a relative fluorescent signal ΔF/F_0_(i,j,t) = (F(i,j,t) - F_0_(i,j)) / F_0_(i,j) was computed. An average was performed over the first 100 ms after stimulus onset to obtain an image of the early evoked response. An image of the late evoked response was similarly obtained by averaging from 100 to 200 ms after stimulus onset. Centers of wS1 and tjS1 were obtained by finding the coordinates of the peak amplitude in the smoothed (sigma = 3 pixels) early evoked response images for whisker and tongue stimulation, respectively. Coordinates of wM1 and tjM1 were identified by finding the local maxima in the frontal motor cortical region using the smoothed (sigma = 3 pixels) late response images.

#### Virus injection and serial two-photon microscopy

An AAV1.hSyn.TurboRFP.WRPE.rBG (titer: 6.5x10^13^ GC/ml, AV-1-PV2642, UPenn Vector Core, USA) was diluted 1:10 or 1:30 in Ringer and injected at the center of tjS1 at 800 μm and 400 μm below the dura. In total 80 nL was delivered through a glass pipette (PCR Micropipets 1 – 10 μl, Drummond Scientific Company, USA) with a 21 – 27 μm inner tip diameter. After three weeks of expression, mice were perfused with 4% PFA (32% Paraformaldehyde (formaldehyde) aqueous solution, Electron Microscopy Science, USA diluted in PBS) and post-fixed overnight. After fixation, we embedded the brains in 5% oxidized agarose (Type-I agarose, Merck KGaA, Germany) and covalently cross-linked the brain to the agarose by incubating overnight at 4°C in 0.5 – 1% sodium borohydride (NaBH_4_, Merck KGaA, Germany) in 0.05 M sodium borate buffer. We imaged the brains in a custom-made two-photon serial microscope, which was controlled using MATLAB-based software (ScanImage 2017b, Vidrio Technologies, USA) and BakingTray https://github.com/BaselLaserMouse/BakingTray, extension for serial sectioning) (Han et al., 2018). The setup consists of a two-photon microscope coupled with a vibratome (VT1000S, Leica, Germany) and a high-precision X/Y/Z stage (X/Y: V-580; Z: L-310, Physik Instrumente, Germany). The thickness of a physical slice was set to be 50 μm for the entire brain and we acquired optical sections at 5 μm using a high-precision piezo objective scanner (PIFOC P-725, Physik Instrumente, Germany) in two channels (green channel: 500 – 550 nm, ET525/50, Chroma, USA; red channel: 580 – 630 nm, ET605/70, Chroma, USA). Each section was imaged by 7% overlapping 1025x1025-μm tiles. A 16x water immersion objective lens (LWD 16x/0.80W; MRP07220, Nikon, Japan), with a resolution of 0.8 μm in X and Y and measured axial point spread function of ∼5 μm full width at half maximum. After image acquisition, the raw images were stitched using a MATLAB-based software (StitchIt, https://github.com/SainsburyWellcomeCentre/StitchIt). This software applies illumination correction based on the average tile in each channel and optical plane and subsequently stitches the tiles from the entire brain. After stitching and before further image processing, we down-sampled the stitched images by a factor of 6 in X and Y obtaining a voxel size of 4.8 × 4.8 × 5 μm, using a MATLAB-based software (MaSIV, https://github.com/SainsburyWellcomeCentre/masiv). To obtain the location of the injection site and the axonal innervation in the frontal part of the cortex we cropped the images at the level of the left cortex using the software Fiji (https://imagej.net/Fiji). To compare the maps to *in vivo* measurements, a 30° rotation was then applied before we resliced the imaged stack from top to bottom. The resulting stack of images was filtered with a Gaussian function (size of kernel was 10 × 10 × 5 pixels) and an average image was created by projecting the fluorescence signal across cortical depth (see Figure S1C for an example without filtering). The global maximum in this image corresponds to the injection site and the second local maximum in the front indicated axonal projections from tjS1. The injection site and the midline were used to align the maps over mice.

#### Pharmacological inactivation

Pharmacological inactivation of cortex was performed by using the GABA receptor agonist muscimol (5 mM, Biotrend, USA). A small craniotomy was made either above the C2 whisker (based on IOS) or above tjM1 (based on stereotaxic coordinates) a day before the pharmacological intervention started. In total 400 nL (wS1) or 500 nL (tjM1) of muscimol or Ringer was injected in four (wS1) or five (tjM1) depths ranging from 200 – 1000 μm below the pia. This was done using a glass pipette with a tip inner diameter of 21 – 27 μm. Ringer was injected on the first day, followed by muscimol for the second day and finally injection of Ringer for the third day.

#### Two-photon imaging

A custom made two-photon setup was built to perform functional calcium imaging. The scanning system consisted of a galvo-resonance mirror pair (8 kHz CRS, Cambridge Technology, USA). The genetically encoded calcium indicator GCaMP6f expressed in Thy1-GCaMP6f mice (Dana et al., 2014) was excited with a 940 nm laser beam coming from a tuneable infrared laser (InSight DeepSee, Spectra Physics - Newport, USA). The acquisition and imaging hardware (NI PXIe-1073, NI PXIe-6341, National Instruments, USA) was controlled by a MATLAB-based software (ScanImage SI5). A GaAsP photosensor module (H10770PA-40, Hamamatsu, Japan) was used to detect emission light after being reflected by two dichroic mirrors (FF705-Di01-25x36 and FF562-Di02-25x36, Semrock, USA) and passing through an infrared blocker (760/SP HC BrightLine, Semrock, USA) and green band pass filter (510/84 BrightLine HC, Semrock, USA). Photocurrents were amplified (DHPCA-100, FEMTO, Germany) and digitized with A/D board (NI 5732 14-bit, NI PXIe-7961R, National Instruments, USA). A Nikon 16x objective (16x Nikon CFI LWD, Japan) and Olympus 40x (40x/0.80 W LUMPLFLN, Japan) were used for prism imaging and a 20x Zeiss (20x W Plan-Apochromat 20x/1,0 DIC, Germany) was used for window imaging. Frame acquisition rate was 30 Hz with resolution of 512x512 pixels.

### Quantification and Statistical Analysis

#### Two-photon calcium data processing

To extract time-varying somatic calcium signals F_Soma_(t), we used the MATLAB-based Suite2p toolbox (Pachitariu et al., 2017) (see Figure S3A for example regions of interests (ROIs)). Neuropil contamination was corrected by subtracting the fluorescent signal from a surrounding ring F_Surround_(t) from somatic fluorescence: F(t) = F_Soma_(t) - α^∗^F_Surround_(t), where α was estimated by the Suite2p deconvolution algorithm and was on average 0.38 ± 0.30. Manual inspection and alignment over sessions was performed by using the built-in toolbox and additional custom-made graphical user interfaces implemented in MATLAB. Rigid alignment was used as offline motion correction for each session. For alignment over sessions, a non-rigid alignment was used, NoRMCorre (Pnevmatikakis and Giovannucci, 2017). Z-scores based on fluorescent traces were computed for each neuron as follow: z-score(t) = (F(t) - F_Mode_) / σ_Noise_, where F_Mode_ and σ_Noise_ are an estimate of the mean noise level and its standard deviation, respectively. The noise level was estimated by finding the mode value for a fluorescence trace (F_Mode_) and by taking the values below the mode and symmetrizing them around the mode to obtain an estimate of standard deviation of the noise (σ_Noise_). This was done in blocks of 100 s. Either the sensory stimulus (auditory or whisker), the first detected lick or small jaw movements were used to align the calcium traces. Neurons that were infrequently active (crossing a threshold defined by 5 standard deviation from baseline calcium activity at rate of less than 0.05 Hz) were discarded. 21% and 22% of the automatically detected and manually curated ROIs showed task dependent modulation for the multisensory detection and multimotor detection task, respectively. To test if a neuron showed a significant response in a given behavioral category (e.g., whisker hit, auditory hit, etc.), we compared baseline activity (for stimulus triggered analysis: −300 – 0 ms; for lick triggered analysis: −800 – −500 ms) with the response window of 0 – 200 ms for stimulus and −100 – 100 ms for lick triggered analysis, respectively (Wilcoxon rank sum test, MATLAB implementation). A minimum of 15 trials per condition for the average calcium response was required. One way to illustrate the population activity is to use principal component analysis (PCA). We took the average calcium responses (stimulus and lick triggered) from −1000 to 3500 ms relative to the analysis trigger for each neuron and pooled them over FOVs. The PCA (MATLAB implementation) was applied over neuron identities. The variance which was explained by the first two components was 76% / 69% (stimulus / lick triggered) and 87% / 83% for the wS1 and tjM1 in multisensory detection task, respectively. In the multimotor task the first two components explained 65% / 56% (stimulus / lick triggered) and 73% / 73% for the wS1 and tjM1, respectively.

#### Lick direction decoder

We implemented a decoder for left versus right licking based on a previously introduced population probabilistic code (Jazayeri and Movshon, 2006, Ma et al., 2006). For each neuron in a given field of view (FOV), we computed the lick-triggered responses to left and right lick in absence of sensory stimuli and outside the reward window (spontaneous licks). Only neurons which showed a significant response to at least one lick direction were considered for the decoder analysis. The significance was tested by comparing the lick triggered responses (−100 – 100 ms) with baseline signals (−700 – −500 ms). At least 15 trials per condition were required for a neuron to be included in the analysis, and a minimum population size of 5 neurons per FOV was imposed. The population activity vector for a single trial consisted of the average response in either the pre-stimulus or post-stimulus window. The pre-stimulus window ranged from −1000 to 0 ms whereas the post-stimulus window went from 0 to 200 ms relative to stimulus onset. The log-likelihood was then computed by doing a matrix multiplication of the population activity vector with logged tuning curves before being summed for all the neurons. We corrected for the response bias in a given FOV by subtraction of the average tuning curves. Finding the lick direction that maximizes the log-likelihood gave us a prediction of the underlying motor action. Appling this procedure to hit and error trials and comparing the decoded motor action with the actual motor action detected on the spouts gave us a decoder performance for single FOVs and sessions.

#### Quantification of small jaw movements

Movies of the face were analyzed with Fiji and MATLAB. A ROI was drawn covering the jaw (see Figure S4). Movements were quantified by averaging the pixel values within the ROI and taking the difference between consecutive frames. The onset time of a small jaw movement was identified as the first time point when change in jaw position was more than two times the standard deviation computed during the pre-stimulus periods (from −2000 to 0 ms relative to stimulus onset) over the entire session. During stimulus trials, only the time period up −500 ms was considered relative to stimulus onset. Two small jaw movements had to be at least 1500 ms apart from each other to be considered as two isolated jaw movements. Spout contacts were defined as licks detected by the piezo-film attached to the spouts.

To quantify the impact of tjM1 and wS1 optogenetic inactivation on jaw movements we extracted the standard deviation from the filmed jaw position (average pixel value within the ROI). The average ratio during the catch window (SD_Catch_) and the preceding baseline window (SD_Baseline_) for correct rejection trials was calculated: SD_Catch_/SD_Baseline_ and compared between light OFF and Light ON trials.

#### Statistics

Data are represented as mean ± SD unless otherwise noted. Wilcoxon signed rank test was used to assess significance in paired comparisons; Wilcoxon rank sum test was used for unpaired comparisons; Pearson correlation coefficient was used to compute correlations between two conditions (MATLAB implementations). The statistical tests used and n numbers are reported explicitly in the main text or figure legends.

### Data and Code Availability

The complete dataset and MATLAB analysis code are freely available at the open access CERN Zenodo database https://zenodo.org/communities/petersen-lab-data with doi hyperlink: https://doi.org/10.5281/zenodo.3271408.

## Supplemental Information

Document S1. Figures S1–S4 and Table S1

Document S2. Article plus Supplemental Information
