# Supplementary material for: Distinct Contributions of Whisker Sensory Cortex and Tongue-Jaw Motor Cortex in a Goal-Directed Sensorimotor Transformation
Source: Neuron. 2019 Sep 25;103(6):1034–1043.e5. doi: 10.1016/j.neuron.2019.07.008 (PMC6859494; doi:10.1016/j.neuron.2019.07.008)
Supplement: Document S1. Figures S1–S4 and Table S1 [file mmc1.pdf]

Neuron, Volume *103*

Supplemental Information

Distinct Contributions of Whisker Sensory Cortex and Tongue-Jaw Motor Cortex in a Goal-Directed Sensorimotor Transformation

Johannes M. Mayrhofer, Sami El-Boustani, Georgios Foustoukos, Matthieu Auffret, Keita Tamura, and Carl C.H. Petersen

Supplemental Information

Distinct contributions of whisker sensory cortex and tongue-jaw motor cortex in a goal-directed sensorimotor transformation

Johannes Mayrhofer, Sami El-Boustani, Georgios Foustoukos, Matthieu Auffret, Keita Tamura and Carl Petersen

Supplemental Information consists of:

Supplemental Figure S1, related to Figure 1

Supplemental Figure S2, related to Figure 2

Supplemental Figure S3, related to Figure 3

Supplemental Figure S4, related to Figure 4

Supplemental Table S1, related to Figure 1

Supplemental Movie S1, related to Figure 1

Supplemental Figure S1

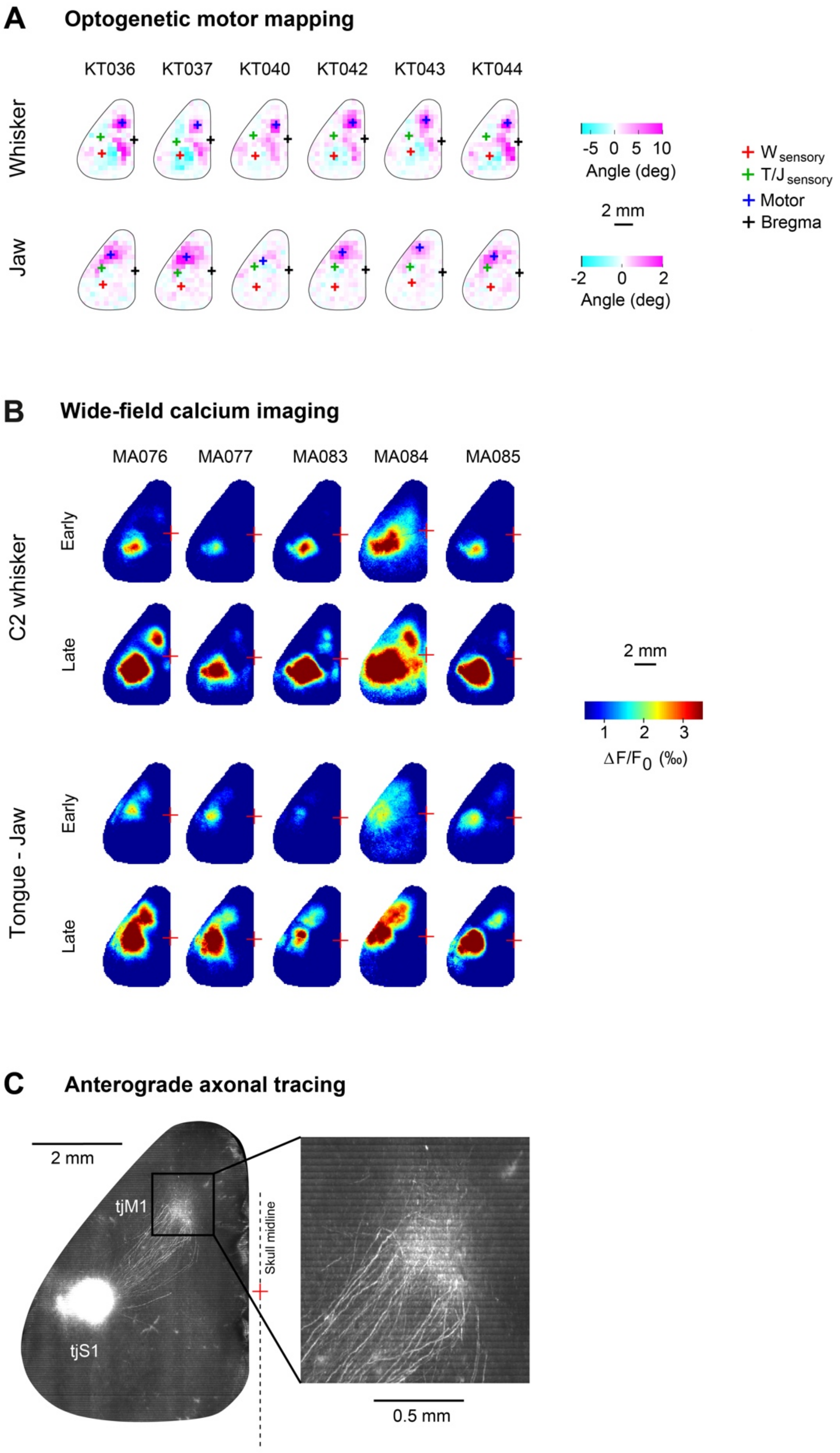

Supplemental Figure S1. Identification of tongue-jaw primary motor cortex, Related to Figure 1

(A) Optogenetic motor mapping. Individual maps for each mouse of whisker and jaw movement amplitudes. Red crosses: center of C2 whisker sensory-evoked intrinsic optical signal. Green crosses: center of tongue sensory intrinsic optical signal. Blue crosses indicate the center of cortical region that produces movement initiation for individual mice. Black crosses: bregma position.

(B) Wide-field calcium imaging. Individual maps for each mouse evoked by whisker and tongue stimulation. The early (0 – 100 ms) response map is plotted above the corresponding late (100 – 200 ms) response map. Red crosses indicate bregma position of individual mice.

(C) Anterograde tracing. AAV was injected into tjS1 and fluorescence imaged across the brain using serial two-photon tomography. Projected fluorescence signal across cortical depth of the rotated and resliced two-photon tomography stack of an example mouse. Red cross indicates location of bregma.

Supplemental Figure S2

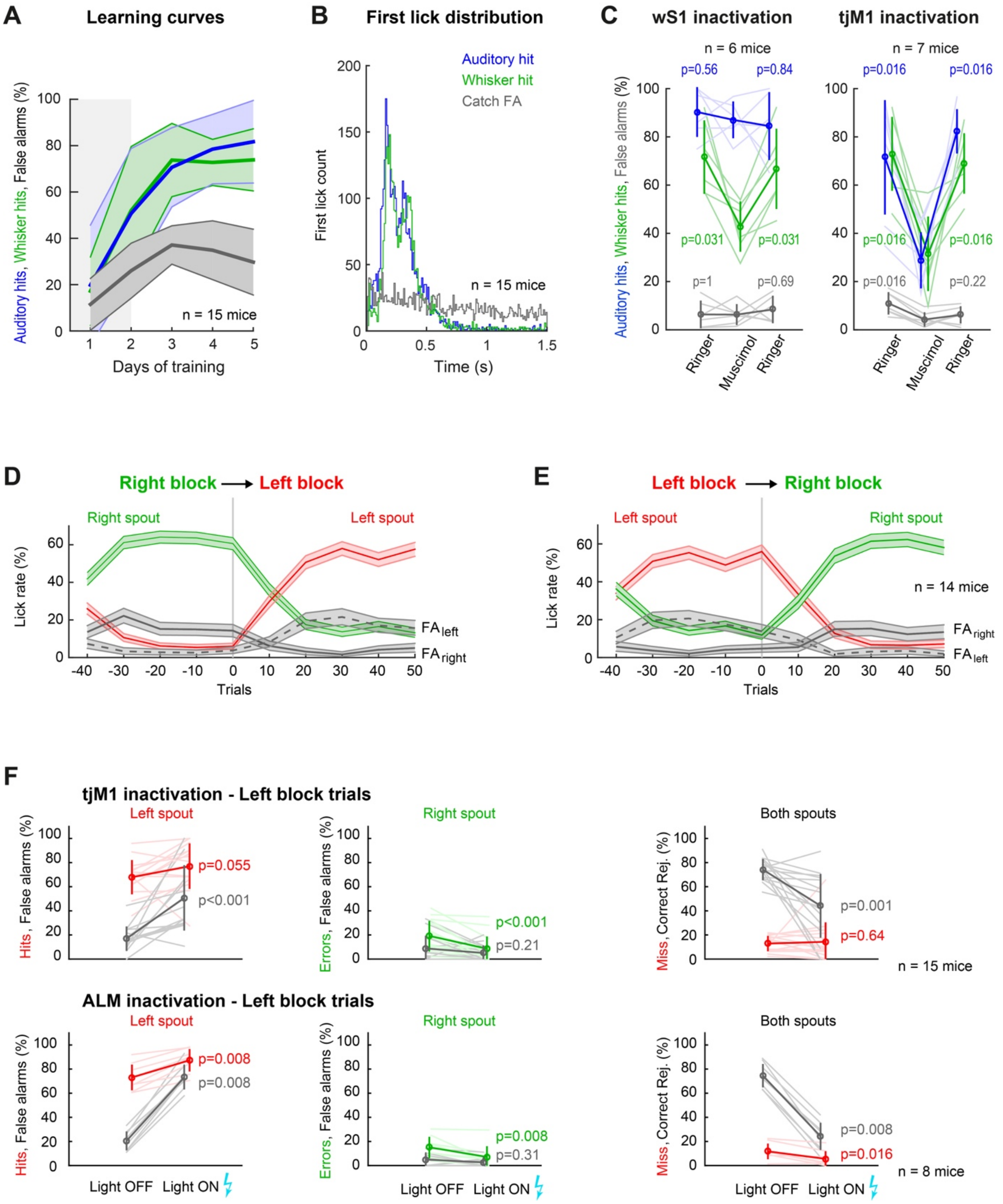

Supplemental Figure S2. Different roles of tjM1 and wS1 during sensorimotor behaviors, Related to Figure 2

(A) Learning curves of two-photon imaged mice. In green the average learning curves for the whisker stimulus and in blue the average learning curves for the auditory stimulus are plotted. The grey shows the false alarm rate over training days.

(B) First lick time distribution for auditory hits (blue), whisker hits (green) and catch false alarms (grey).

(C) Muscimol inactivation experiments in the multisensory detection task. On the first day a vehicle, on the second day a muscimol and the third day another vehicle injection with Ringer was used. Same color code as in panels A and B.

(D and E) Lick probability for right spout (green) and left spout (red) aligned to the rewarded spout switch time (n = 14 mice). The lick probability for the catch (right and left) trials is shown in grey. In panel D, the right spout rewarded to left spout rewarded switch (as shown in Figure 2). In panel E, the complementary switch from left spout rewarded to right spout rewarded is shown.

(F) tjM1 (n = 15 mice) and ALM (n = 8 mice) inactivation during the multimotor task. Upper graphs show the effect of tjM1 inactivation on left and right spout lick probability during left block trials, as well as miss rates. Lower graphs show the same, but during ALM inactivation.

Data are represented as mean ± SD, except in panels D and E, which show 95% confidence intervals. Wilcoxon signed rank test in panels C and F.

Supplemental Figure S3

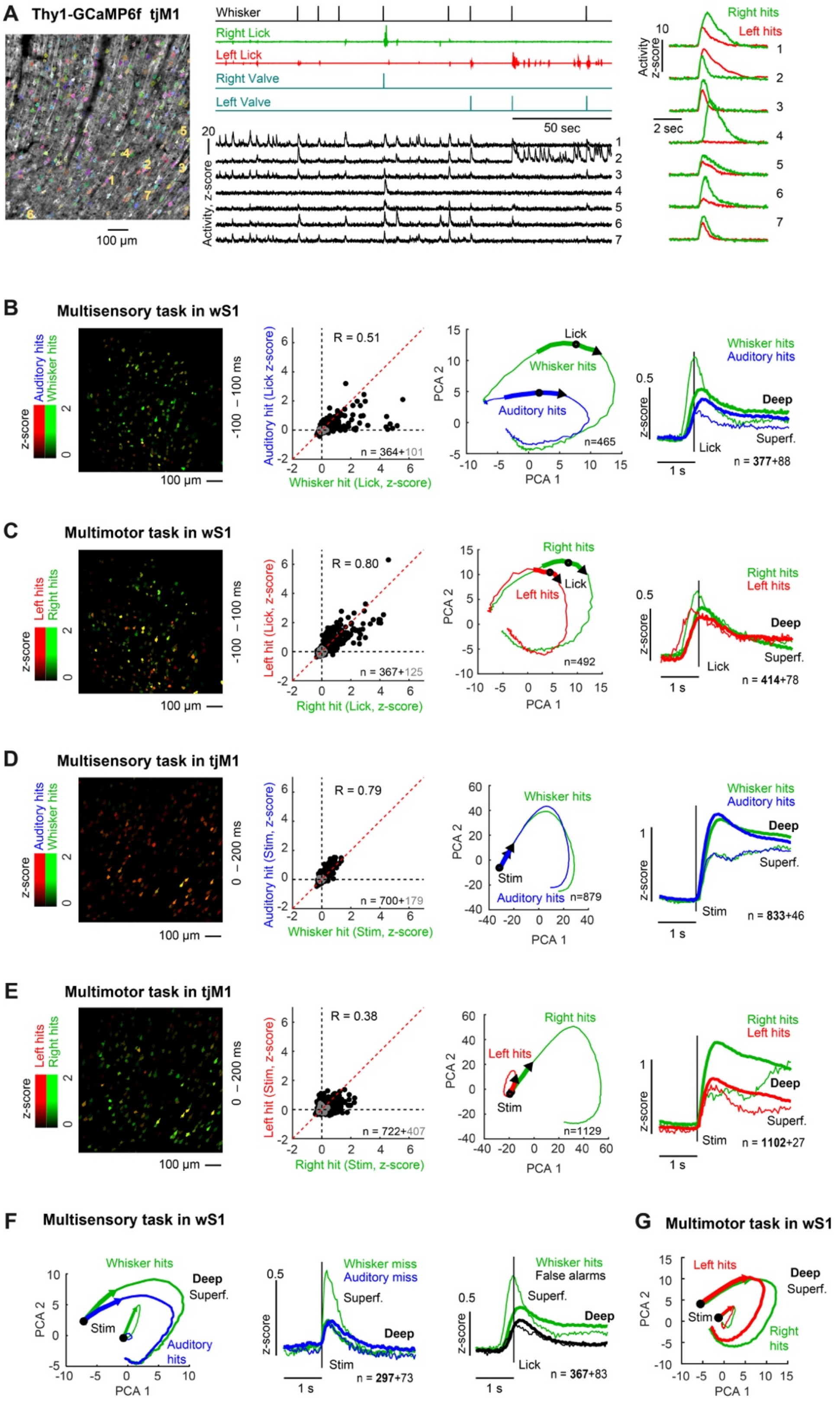

Supplemental Figure S3. Neuronal correlates of multisensory and multimotor decision making, Related to Figure 3

(A) Left: Extracted regions of interest from Suite2P (Pachitariu et al., 2017). The grey scale image shows a time average of the two-photon calcium image in an example tjM1 field of view. The colored regions correspond to different ROIs found by the algorithm. Middle: Traces of behavioral variables and calcium signals from seven example neurons depicted in panel A during the multimotor detection task. Right: Stimulus triggered averages of the cells.

(B) Left: Example field of view (FOV) during a multisensory task in wS1. Red image channel: average lick triggered responses (-100 – 100 ms) for auditory hit trials. Green image channel: same but for whisker hit trials. Middle left: Scatter plot over all neurons comparing average response during whisker and auditory hit trials. Grey points did not show any significant modulation to the shown categories. R is the Pearson correlation coefficient. Middle right: PCA of the time-varying neuronal population vector during whisker and auditory hit trials. Thick lines indicate -100 – 100 ms around first lick. Arrow heads show direction of the trajectories. Right: Grand average whisker and auditory z-score evoked responses in hit trials for deep (thick) and superficial (thin) neurons aligned to lick time.

(C) Left: Example FOV during a multimotor task in wS1. Red image channel: average lick triggered responses (-100 – 100 ms) for left hit trials. Green image channel: same but for right hit trials. Middle left: Scatter plot over all neurons comparing average response during left and right hit trials. Grey points did not show any significant modulation to the shown categories. R is the Pearson correlation coefficient. Middle right: PCA of the time-varying neuronal population vector during left and right hit trials. Thick lines indicate -100 – 100 ms around first lick. Arrow heads show direction of the trajectories. Right: Grand average left and right z-score evoked responses in hit trials for deep (thick) and superficial (thin) neurons aligned to lick time.

(D and E) same as panels B and C, but for recordings performed in tjM1. Average responses were calculated in the time window 0 – 200 ms relative to stimulus onset. Pearson correlation coefficients in panels B – E: $p < 0.001$.

(F) Left: PCA for Deep (Thick) and Superficial (Thin) neurons in wS1 of the time-varying neuronal population vector during whisker and auditory hit trials. Lines with arrowheads indicate the first 200 ms after stimulus onset. Middle: Grand average whisker and auditory z-score evoked responses in miss trials for deep (thick) and superficial (thin) neurons aligned to stim time. The average z-score response for whisker misses was 0.28 ± 0.44 compared to auditory misses 0.19 ± 0.22; $p = 0.14$, Wilcoxon signed rank test; $n = 271$). Separately analyzing superficial and deep neurons, we found: i) Superficial neurons: average z-score response for whisker miss 0.42 ± 0.65 vs auditory miss 0.14 ± 0.21, $p = 0.001$, $n = 62$; ii) Deep neurons: average z-score response for whisker miss 0.23 ± 0.34 vs auditory miss 0.20 ± 0.23, $p = 0.87$, $n = 209$; Wilcoxon signed rank tests. The difference in whisker miss and auditory miss response was significantly larger for superficial compared to deep neurons ($p < 0.001$ Wilcoxon rank sum test). Right: Grand average whisker hit and false alarm z-score responses for deep (thick) and superficial (thin) neurons aligned to lick time. False alarm licking gave rise to an average z-score increase of 0.24 ± 0.39, aligned to lick onset (-100 to 100 ms relative to lick time), compared to the whisker-evoked licking which had an average z-score increase of 0.53 ± 0.82 ($p < 0.001$, Wilcoxon signed rank test, $n = 348$), aligned to lick onset. In superficial wS1 neurons we found an average z-score response in whisker hit trials of 0.95 ± 1.19 and in false alarm trials 0.26 ± 0.29 ($p < 0.001$, Wilcoxon signed rank test; $n = 66$). The superficial neurons responded with a ~4 times larger response in whisker hit compared to false alarm trials. In deep wS1 neurons we found an average z-score response in whisker hit trials of 0.43 ± 0.68 and in false alarm trials 0.23 ± 0.41 ($p < 0.001$, Wilcoxon signed rank test; $n = 282$). The deep neurons responded with ~2 times larger response in whisker hit compared to false alarm trials. The superficial neurons therefore appear to be more strongly modulated by sensory input compared to the deep neurons, and the difference between whisker hits and false alarms was significantly larger in superficial neurons compared to deep neurons ($p < 0.001$, Wilcoxon rank sum test).

(G) PCA for Deep (Thick) and Superficial (Thin) neurons in wS1 of the time-varying neuronal population vector during left and right hit trials. Lines with arrowheads indicate the first 200 ms after stimulus onset.

Supplemental Figure S4

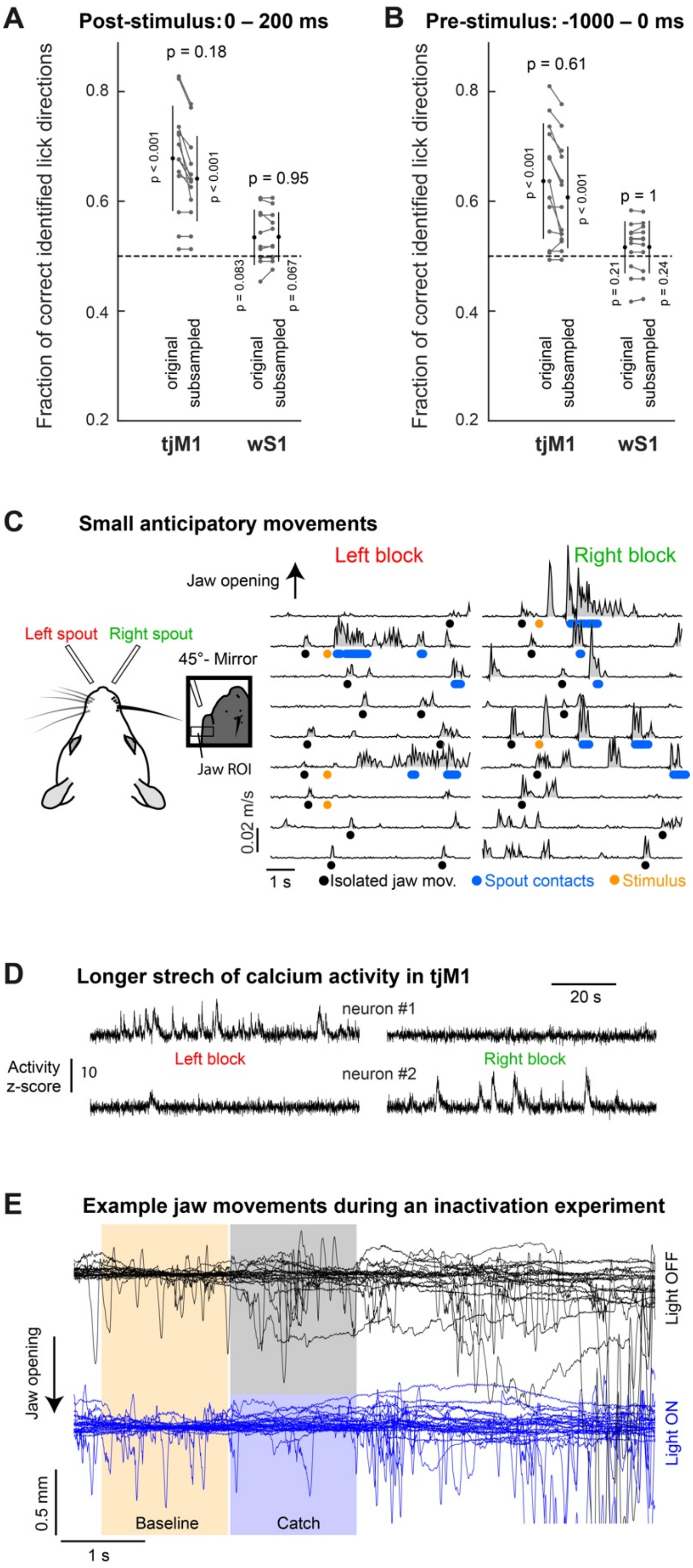

Supplemental Figure S4. Decoding of lick direction and small anticipatory jaw movements, Related to Figure 4

(A) Subsampled result for decoder performance for the post-stimulus analysis window. We restricted the decoder population size of the individual FOVs to a maximum of 30 neurons, which is the average wS1 decoder population size (30.5 ± 17.7 neurons in wS1 and 68.2 ± 44.0 neurons in tjM1). This led to an average population size for the decoder of 23.9 ± 10.6 neurons in wS1 and of 25.2 ± 9.6 neurons in tjM1. Left columns correspond to the original data shown in Figure 4B. Right columns are the averages of 1000 subsampled results for each FOV. Wilcoxon signed rank test was used to test against chance (dashed line); Wilcoxon rank sum test was used to test between groups.

(B) Subsampled result for decoder performance for pre-stimulus analysis window. Same as panel A, but for pre-stimulus window.

(C) Extraction of small jaw movements outside the stimulus window and spout contacts. Only onsets of small jaw movements which were more than 500 ms before a whisker stimulus and more than 1500 ms from the last small jaw movement were considered. In addition, only onsets which occurred 500 ms after filming started were selected.

(D) Long stretch of calcium activity of two tjM1 example neurons during left and right block trials.

(E) Example jaw movements during an inactivation experiment. Black traces are correct rejection trials without light. Blue traces correspond to correct rejection trials with light. Individual traces were baseline aligned. Shaded areas represent analysis windows: light ON (blue), light OFF (grey) and baseline (yellow) periods.

Supplemental Table S1. Summary table of mapping experiments, Related to Figure 1

A Optogenetic motor mapping

mm	Bregma		wS1 (IOS)		wM1		tjS1 (IOS)		tjM1	
	M-L	A-P	M-L	A-P	M-L	A-P	M-L	A-P	M-L	A-P
KT036	0.0	0.0	3.47	-1.47	1.30	1.86	3.61	0.31	2.62	1.73
KT037	0.0	0.0	3.34	-1.68	1.45	1.64	3.72	-0.21	2.76	1.53
KT040	0.0	0.0	3.50	-1.82	1.23	1.55	3.71	0.38	2.75	0.97
KT042	0.0	0.0	3.32	-1.85	1.30	1.75	3.33	0.31	2.51	1.91
KT043	0.0	0.0	3.31	-1.13	1.67	2.29	3.51	0.58	2.45	2.64
KT044	0.0	0.0	3.09	-1.52	1.18	2.05	3.47	0.57	2.73	1.76
Average	**0.0**	**0.0**	**3.34**	**-1.58**	**1.35**	**1.86**	**3.56**	**0.32**	**2.64**	**1.76**
SD	0.0	0.0	0.14	0.27	0.18	0.28	0.15	0.29	0.13	0.54

B Wide-field calcium imaging

mm	Bregma		wS1		wM1		tjS1		tjM1	
	M-L	A-P	M-L	A-P	M-L	A-P	M-L	A-P	M-L	A-P
MA076	0.0	0.0	3.81	-1.40	1.61	1.40	4.01	0.30	2.81	1.80
MA077	0.0	0.0	3.68	-1.39	1.68	1.81	4.08	-0.09	2.68	1.91
MA083	0.0	0.0	3.24	-1.10	1.24	1.30	3.64	0.40	2.74	1.90
MA084	0.0	0.0	3.29	-1.59	1.19	1.11	3.89	-0.39	2.69	1.71
MA085	0.0	0.0	3.05	-1.39	0.65	1.71	3.35	-0.29	1.15	1.71
Average	**0.0**	**0.0**	**3.42**	**-1.38**	**1.28**	**1.47**	**3.80**	**-0.01**	**2.42**	**1.81**
SD	0.0	0.0	0.28	0.16	0.37	0.26	0.27	0.31	0.63	0.09

C Anterograde axonal tracing

mm	Bregma		tjS1		tjM1	
	M-L	A-P	M-L	A-P	M-L	A-P
MA077	0.0	0.0	4.12	-0.20	2.02	1.76
MA078	0.0	0.0	3.87	0.00	2.30	1.46
MA083	0.0	0.0	3.64	0.60	2.10	1.70
MA084	0.0	0.0	3.40	0.00	1.99	1.33
MA085	0.0	0.0	3.90	-0.20	2.24	1.20
Average	**0.0**	**0.0**	**3.79**	**0.04**	**2.13**	**1.49**
SD	0.0	0.0	0.28	0.33	0.13	0.24

Supplemental Movie S1. Image stack using serial two-photon tomography, Related to Figure 1

A movie of serial coronal sections from injection site (tjS1) to anterior slices where axons innervate the motor cortex (tjM1). The data are downsampled by a factor of 6 for X and Y, and by a factor of 10 for Z.
